# A polarization-insensitive plasmonic photoconductive terahertz emitter

**DOI:** 10.1063/1.5006273

**Published:** 2017-11-16

**Authors:** Xurong Li, Nezih Tolga Yardimci, Mona Jarrahi

**Affiliations:** Electrical Engineering Department, University of California – Los Angeles, Los Angeles, California 90095, USA

## Abstract

We present a polarization-insensitive plasmonic photoconductive terahertz emitter that uses a two-dimensional array of nanoscale cross-shaped apertures as the plasmonic contact electrodes. The geometry of the cross-shaped apertures is set to maximize optical pump absorption in close proximity to the contact electrodes. The two-dimensional symmetry of the cross-shaped apertures offers a polarization-insensitive interaction between the plasmonic contact electrodes and optical pump beam. We experimentally demonstrate a polarization-insensitive terahertz radiation from the presented emitter in response to a femtosecond optical pump beam and similar terahertz radiation powers compared to previously demonstrated polarization-sensitive photoconductive emitters with plasmonic contact electrode gratings at the optimum optical pump polarization.

Photoconductive emitters are one of the most promising sources of terahertz radiation.^1–10^ They are comprised of an ultrafast photoconductor integrated with a terahertz radiating element. When an optical pump beam with terahertz frequency components is incident of the photoconductor, a terahertz photocurrent is induced and coupled to the terahertz radiating element to generate terahertz radiation. Since the first demonstration of photoconductive emitters in the 1980s,^1^ numerous efforts have been made to enhance their output power, efficiency, and bandwidth.

It was recently shown that incorporating plasmonic contact electrodes in photoconductive emitters enhances their terahertz radiation power significantly.^11–24^ This enhancement is due to the excitation of surface plasmon waves near the metal contact-substrate interface, which enhances the concentration of the photo-generated carriers in close vicinity to the contact electrodes. By reducing the average transport path length of the photo-generated carriers to the contact electrodes, a significantly larger number of photo-generated carriers drifts to the terahertz radiating element in a sub-picosecond time-scale, and higher terahertz power levels are generated. By using two-dimensional (2D) and three-dimensional (3D) plasmonic contact electrode gratings up to 2 orders-of-magnitude and 3 orders-of-magnitude enhancement in the optical-to-terahertz conversion efficiency of photoconductive emitters have been demonstrated, respectively. However, performance of the previously demonstrated plasmonic photoconductive emitters has been very sensitive to the polarization state of the optical pump beam due to the use of grating-based plasmonic contact electrodes.^11^ This polarization sensitivity is a major problem for future terahertz imaging, spectroscopy, and communication systems based on plasmonic photoconductive emitters integrated with compact, lightweight, and low cost light sources.^18,19^ Polarization variability is a direct outcome of using semiconductor lasers (e.g., VCSELs) and fiber lasers fabricated at high volumes and utilized in field settings at which external thermal and mechanical stimuli are inevitable. Therefore, polarization-insensitive plasmonic contact electrode structures are in high demand to maintain high optical-to-terahertz conversion efficiencies under various optical polarization conditions.

In this work, we present a polarization-insensitive plasmonic photoconductive terahertz emitter, which utilizes a 2D array of nanoscale cross-shaped apertures as the plasmonic contact electrodes. The 2D symmetry of the cross-shaped apertures leads to a polarization-insensitive interaction between the plasmonic contact electrodes and optical pump beam. We demonstrate a polarization-insensitive terahertz radiation power from the presented photoconductive emitter, which generates similar terahertz radiation powers compared with previously demonstrated photoconductive emitters with plasmonic contact electrode gratings at the optimum optical pump polarization.

Figure 1(a) shows the schematic diagram of the polarization-insensitive plasmonic photoconductive emitter, which is fabricated on a semi-insulating GaAs (SI-GaAs) substrate. A logarithmic spiral antenna is used as the terahertz radiating element. The antenna is designed to offer a broadband radiation resistance of 70-100 Ω and a reactance near 0 Ω over the 0.1-2 THz frequency range.^25,26^ A 2D array of cross-shaped metallic apertures is used as the polarization-insensitive plasmonic contact electrodes. Their 2D symmetry offers a polarization-insensitive interaction between the optical pump beam and the plasmonic contact electrodes. Compared to other 2D symmetric subwavelength apertures (circular-shaped or square-shape), cross-shaped apertures offer higher optical transmission into the substrate.^27^ A 45 nm thick Au layer with a 3 nm Ti adhesion layer is used to form the cross-shaped apertures, and a 340 nm thick silicon nitride layer is used as an anti-reflection coating. The geometry of the cross-shaped apertures (periodicity = 200 nm, length = 150 nm, width = 50 nm) is chosen to maximize optical absorption in a 100 nm-deep GaAs region beneath the contact electrodes at an 800 nm wavelength.

**FIG. 1.**
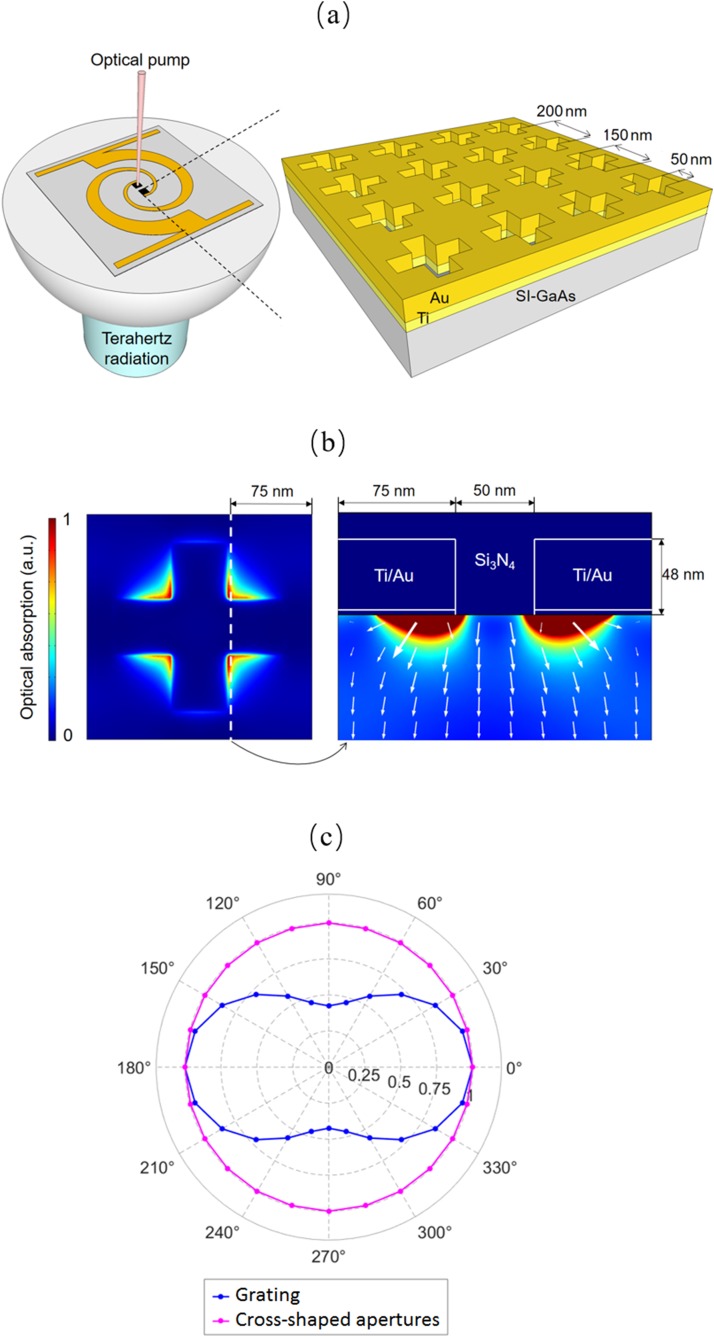
(a) Schematic diagram of the polarization-insensitive plasmonic photoconductive emitter with cross-shaped aperture array contact electrodes fabricated on a SI-GaAs substrate. (b) Top view color plot of optical absorption inside the GaAs substrate at a 1 nm depth below its surface (left) and cross-sectional view color plot of optical absorption inside the GaAs substrate (right) at an optical pump wavelength of 800 nm. The white arrows show the optical power flow direction. (c) Normalized optical absorption in a 100 nm-deep GaAs region beneath the designed cross-shaped plasmonic contact electrode (purple data) and a plasmonic contact electrode grating (blue data) as a function of the optical pump polarization.

A finite-element method based software package (COMSOL) is used to analyze the interaction of an 800 nm optical pump beam with the designed cross-shaped aperture arrays. The analysis shows that the excitation of surface plasmon waves on the cross-shaped aperture arrays bends the direction of the transmitted optical pump beam through the apertures and enhances the photocarrier concentration near the contact electrode-substrate interface (Fig. 1(b)). The analysis also shows that the optical transmission through the cross-shaped plasmonic contact electrodes and the photocarrier concentration in the substrate are not affected by the polarization state of the optical pump beam. Figure 1(c) compares the optical absorption in a 100 nm-deep GaAs region beneath the designed cross-shaped plasmonic contact electrode with that of a plasmonic contact electrode grating. The geometry of the plasmonic contact electrode grating (3 nm Ti/45 nm Au, periodicity = 225 nm, width = 125 nm) is chosen to maximize optical absorption in a 100 nm-deep GaAs region beneath the contact electrodes for an 800 nm optical pump beam polarized normal to the gratings.^12^ While the optical absorption beneath the cross-shaped plasmonic contact electrode remains constant at all optical pump polarizations, it varies significantly for the plasmonic grating. Our analysis estimates an ∼2.5 times reduction in optical absorption beneath the plasmonic grating (∼6 times reduction in the generated terahertz power level) when the optical pump polarization direction is changed from the normal to parallel orientation relative to the grating.

Prototypes of the designed polarization-insensitive photoconductive emitter with cross-shaped plasmonic contact electrodes are fabricated on a SI-GaAs substrate. The microscope image of a fabricated plasmonic photoconductive emitter and the scanning electron microscope (SEM) image of the utilized cross-shaped plasmonic contact electrode are shown in Fig. 2(a). Two cross-shaped plasmonic contact electrodes are connected to the two input arms of the logarithmic spiral antenna. The cross-shaped plasmonic contact electrodes have a 20 × 20 μm^2^ area and are separated by a 10 μm gap. The fabrication process began with patterning the cross-shaped aperture arrays using electron-beam lithography, followed by deposition of Ti/Au (3/45 nm) and liftoff. Then, the antenna and bias lines were formed using optical lithography, Ti/Au (20/400 nm) deposition, and liftoff. A 340 nm thick silicon nitride coating was added by plasma-enhanced chemical vapor deposition. Next, the contact vias were opened using optical lithography and dry plasma etching. Finally, the device was mounted on a silicon lens and placed on a rotation mount.

**FIG. 2.**
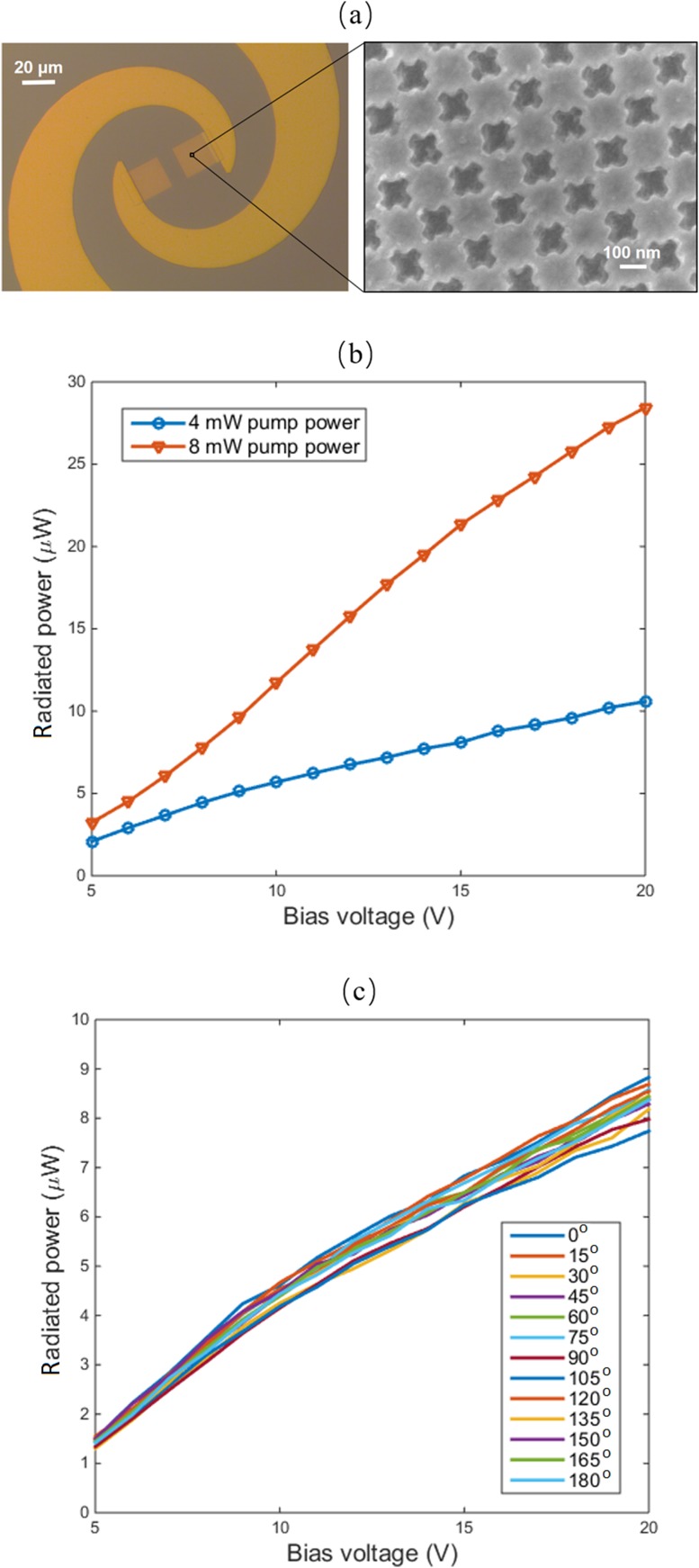
(a) Microscope image of the fabricated plasmonic photoconductive emitter and the SEM image of the cross-shaped plasmonic contact electrodes. (b) Radiation power from the fabricated terahertz emitter as a function of the bias voltage and optical pump power. (c) Radiation power from the fabricated terahertz emitter at different optical pump polarization angles for a 4 mW optical pump power.

The fabricated plasmonic photoconductive emitter is characterized in response to 135 fs optical pump pulses from a Ti:sapphire mode-locked laser with a central wavelength of 800 nm and a repitition rate of 76 MHz. The optical pump beam is first transmitted through a quarter-wave plate (Thorlabs, AQWP05M-950) and a polarization rotator (Thorlabs, LPVIS050-MP) for controlling the polarization angle. The optical pump beam is then focused onto the anode plasmonic contact electrode to maximize the induced ultrafast photocurrent and optimize the impedance loading to the logarithmic spiral antenna.^28,29^ The terahertz radiation power is measured using a calibrated pyroelectric detector (Spectrum Detector Inc., SPI-A-65 THz). Figure 2(b) shows the measured radiation power as a function of the optical pump power and bias voltage. A terahertz radiation power of 28.4 μW is measured at an 8 mW optical pump power and a bias voltage of 20 V, which is comparable with the power of the previously demonstrated grating-based plasmonic photoconductive emitters with identical logarithmic spiral antennas at the optimum optical pump polarization.^12^ The terahertz radiation power from the fabricated plasmonic photoconductive emitter is measured at different optical pump polarization angles, while maintaining a 4 mW optical pump power. Unlike grating-based plasmonic photoconductive emitters,^11^ a negligible variation in the terahertz radiation power is observed when varying the optical polarization angle from 0° to 180° (Fig. 2(c)), offering a polarization insensitive solution for practical applications.

The radiation field from the fabricated plasmonic photoconductive emitter with cross-shaped contact electrodes is characterized at a 4 mW optical pump power using a time-domain terahertz spectroscopy setup with a plasmonic photoconductive terahertz detector based on nano-antenna arrays.^30^ The time-domain radiated field and the frequency-domain radiation power, calculated by taking the fast Fourier transforms of the radiation field, are shown in Figs. 3(a) and (b), respectively.

**FIG. 3.**
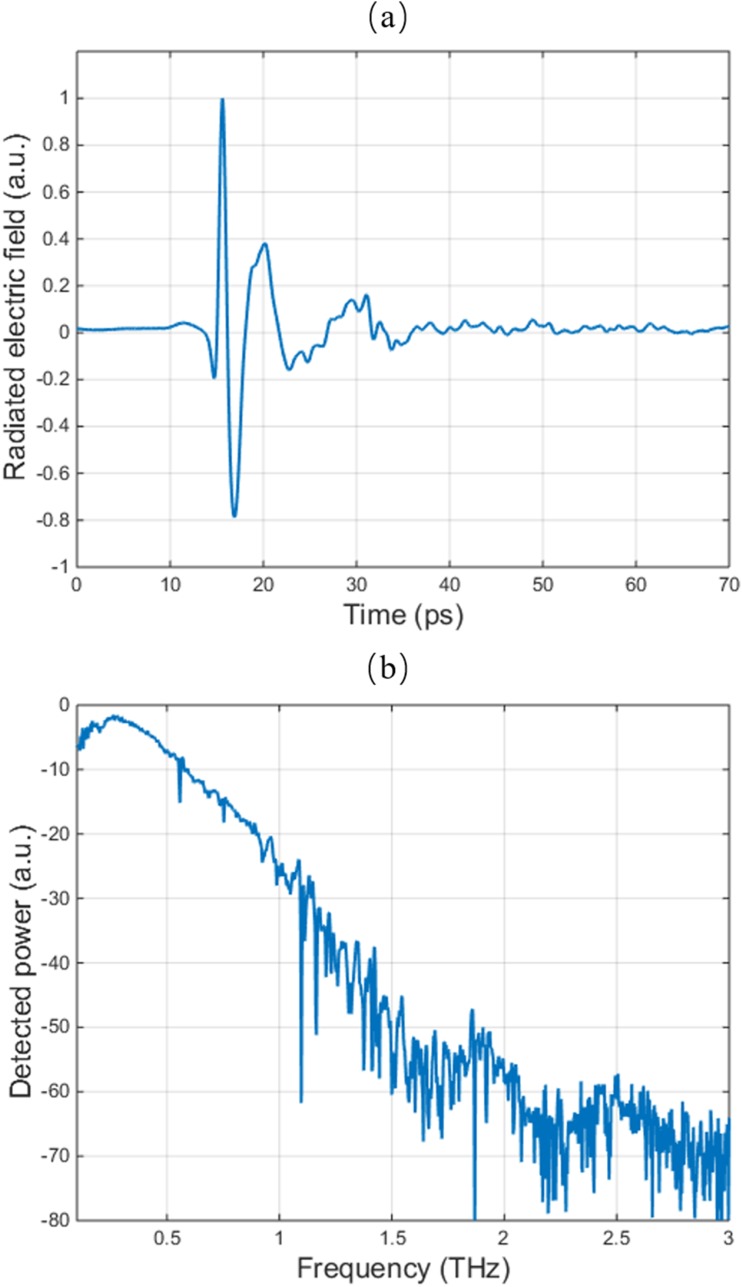
(a) Radiated electric field in the time-domain and (b) radiation power in the frequency-domain from the plasmonic photoconductive emitter with cross-shaped contact electrodes.

In summary, we demonstrate a polarization-insensitive plasmonic photoconductive terahertz emitter based on cross-shaped plasmonic contact electrodes. The 2D symmetry of the cross-shaped aperture arrays leads to the same optical pump absorption and terahertz radiation power at different optical pump polarization angles without any sacrifice in the terahertz radiation power. The terahertz radiation power from the demonstrated polarization-insensitive plasmonic photoconductive emitter is comparable to previously demonstrated grating-based plasmonic photoconductive emitters at their optimum optical pump polarization angle. The use of 2D symmetric plasmonic contact electrodes is not limited to the demonstrated photoconductive emitter and can be extended to other terahertz emitter architectures operating at different optical pump wavelengths.
